# A Novel Deep Learning Model for Predicting Colorectal Anastomotic Leakage: A Pioneer Multicenter Transatlantic Study

**DOI:** 10.3390/jcm14155462

**Published:** 2025-08-03

**Authors:** Miguel Mascarenhas, Francisco Mendes, Filipa Fonseca, Eduardo Carvalho, Andre Santos, Daniela Cavadas, Guilherme Barbosa, Antonio Pinto da Costa, Miguel Martins, Abdullah Bunaiyan, Maísa Vasconcelos, Marley Ribeiro Feitosa, Shay Willoughby, Shakil Ahmed, Muhammad Ahsan Javed, Nilza Ramião, Guilherme Macedo, Manuel Limbert

**Affiliations:** 1 Precision Medicine Unit, Department of Gastroenterology, Unidade Local de Saúde São João, 4200-319 Porto, Portugal; francisco.cnm@gmail.com (F.M.); miguelpedro96@gmail.com (M.M.); guilhermemacedo59@gmail.com (G.M.); 2 WGO Gastroenterology and Hepatology Training Center, 4200-427 Porto, Portugal; 3 Faculty of Medicine, University of Porto, 4200-427 Porto, Portugal; andre.francisco.martins@gmail.com; 4 CINTESIS-RISE, Department of Community Medicine, Information and Health Decision Sciences (MEDCIDS), Faculty of Medicine, University of Porto, 4200-427 Porto, Portugal; 5 DigestAID, Digestive Artificial Intelligence Development, 4200-135 Porto, Portugal; antoniompintodacosta@gmail.com; 6 Instituto Português de Oncologia de Lisboa, 1099-023 Lisboa, Portugal; filipa.arfonseca@gmail.com (F.F.); manuellimbert@gmail.com (M.L.); 7 INEGI—Instituto de Ciência e Inovação em Engenharia Mecânica e Engenharia Industrial, 4200-465 Porto, Portugal; ecarvalho@inegi.up.pt (E.C.); nramiao@inegi.up.pt (N.R.); 8 Faculty of Engineering, University of Porto, 4200-427 Porto, Portugal; 9 Hospital Universitario Puerta de Hierro Majadahonda, 28222 Madrid, Spain; 10 Royal Liverpool University Hospital, Liverpool L7 8YE, UK; abdullah.bunaiyan@liverpoolft.nhs.uk (A.B.); shay.willoughby@liverpoolft.nhs.uk (S.W.); ahsanj@liverpool.ac.uk (M.A.J.); 11 Hospital das Clinicas da Faculdade de Medicina da Universidade de São Paulo, Clínica de Ribeirao Preto, Ribeirao 05403-010, Brazilmrfeitosa@hcrp.usp.br (M.R.F.)

**Keywords:** artificial intelligence, deep learning, anastomosis leakage

## Abstract

**Background/Objectives**: Colorectal anastomotic leak (CAL) is one of the most severe postoperative complications in colorectal surgery, impacting patient morbidity and mortality. Current risk assessment methods rely on clinical and intraoperative factors, but no real-time predictive tool exists. This study aimed to develop an artificial intelligence model based on intraoperative laparoscopic recording of the anastomosis for CAL prediction. **Methods**: A convolutional neural network (CNN) was trained with annotated frames from colorectal surgery videos across three international high-volume centers (Instituto Português de Oncologia de Lisboa, Hospital das Clínicas de Ribeirão Preto, and Royal Liverpool University Hospital). The dataset included a total of 5356 frames from 26 patients, 2007 with CAL and 3349 showing normal anastomosis. Four CNN architectures (EfficientNetB0, EfficientNetB7, ResNet50, and MobileNetV2) were tested. The models’ performance was evaluated using their sensitivity, specificity, accuracy, and area under the receiver operating characteristic (AUROC) curve. Heatmaps were generated to identify key image regions influencing predictions. **Results**: The best-performing model achieved an accuracy of 99.6%, AUROC of 99.6%, sensitivity of 99.2%, specificity of 100.0%, PPV of 100.0%, and NPV of 98.9%. The model reliably identified CAL-positive frames and provided visual explanations through heatmaps. **Conclusions**: To our knowledge, this is the first AI model developed to predict CAL using intraoperative video analysis. Its accuracy suggests the potential to redefine surgical decision-making by providing real-time risk assessment. Further refinement with a larger dataset and diverse surgical techniques could enable intraoperative interventions to prevent CAL before it occurs, marking a paradigm shift in colorectal surgery.

## 1. Introduction

Anastomotic leaks (ALs) remain among the most serious and feared complications following intestinal surgery, particularly in the colorectal oncologic setting [1,2,3]. They are associated with increased morbidity, prolonged hospitalization, delays in adjuvant therapy, and worsened long-term oncologic outcomes and quality of life [4,5]. The International Study Group of Rectal Cancer defines AL as a defect in the integrity of the intestinal wall at the anastomotic site, resulting in communication between intra- and extraluminal compartments [6]. ALs are classified retrospectively based on their clinical severity and the interventions required, but this system is only applicable once the complication has fully declared itself, limiting its value in early or preventive strategies [6]. Considering the economic impact, anastomotic leakage (AL) was associated with an incremental cost of up to EUR 80,000 per patient, with the majority of expenses related to operating room use, ICU admission, medications, and follow-up consultations [7]. Including the estimated societal burden, a study reported an annual cost of EUR 6.1 million in the United Kingdom [8].

Despite ongoing advances in surgical techniques and perioperative management, early and reliable identification of AL remains a clinical challenge [9]. Although the conventional surgical understanding indicates that most ALs occur between postoperative days 5 and 7, some are detected later, even after hospital discharge [10,11]. Maintaining a high index of suspicion is crucial for the early detection of AL, promoting a timely medical intervention and improving treatment outcomes [12].

Conventional diagnostic approaches—such as clinical assessment, biochemical markers, and imaging—are typically employed reactively only after the onset of symptoms and are heavily reliant on clinical suspicion and the surgeon’s judgment [13]. However, early clinical signs of AL are often subtle, are non-specific, and may overlap with other postoperative complications [14]. Classical signs are neither pathognomonic nor consistently present, underscoring the importance of sustained clinical vigilance and the limitations of relying solely on conventional diagnostic modalities [15]. Any unexpected change in a patient’s postoperative recovery should prompt further evaluation [9]. To address these diagnostic limitations, clinicians frequently turn to laboratory markers such as leukocyte counts, C-reactive protein (CRP), and procalcitonin (PCT), which, although helpful in ruling out ALs due to their high negative predictive value, are less effective in predicting their occurrence [15]. Blood tests offer a cost-effective method to either raise or lower the level of suspicion for CAL, making them a useful initial step in the diagnostic process [16]. In the presence of clinical suspicion, these laboratory findings are often complemented by imaging studies, mostly computed tomography (CT) scans and water-soluble contrast enemas [17]. However, these modalities are also limited by suboptimal sensitivity, inter-observer variability, and logistical challenges such as availability [18]. Radiologic signs of AL, such as fluid or gas collections adjacent to the anastomosis, are not always definitive, and contrast extravasation may be absent [19]. There is considerable overlap in CT signs between patients with and without a significant leak, emphasizing the need to interpret imaging findings alongside clinical symptoms [15]. When laboratory and imaging findings are inconclusive, endoscopic evaluation may offer valuable diagnostic insight [16]. Endoscopy can offer direct visualization of the anastomosis, as well as the overall condition of the area, including signs of necrosis, ischemia, indentation, and the orientation of the anastomotic line [20]. However, this approach is costly and time-consuming, carries procedural risks, and may not be readily accessible [16].

Collectively, these modalities form a diagnostic pathway that is inherently reactive, with investigations typically initiated only after the patient becomes symptomatic [21]. This reactive approach limits the opportunity for early intervention or even preventive intraoperative interventions [22]. What remains lacking is an objective, real-time intraoperative method to assess anastomotic integrity and stratify risk before clinical deterioration occurs.

Intraoperative assessment of anastomosis integrity is a critical moment in colorectal surgery, but it remains largely subjective [22]. Traditional techniques—such as visual inspection, air leak testing, perfusion assessment with or without indocyanine green (ICG), fluorescence angiography, and, in selected cases, endoscopic inspection—aim to identify technical or perfusion-related issues that could compromise healing [22]. However, these techniques offer limited predictive accuracy and are influenced by surgeon experience and interpretation [23]. As such, high-risk anastomoses may go undetected, even in seemingly uneventful procedures [24].

The growing adoption of minimally invasive surgery (MIS) has not only enhanced visualization and reduced complication rates but also enabled the routine capture of high-definition surgical video [25]. These digital records are more than just quality assurance tools—they represent a rich data source for the development of intraoperative, AI-powered decision support systems [26,27].

Artificial intelligence (AI) is emerging as a transformative technology in surgical decision-making and research. Supervised learning models have shown potential in recognizing key anatomical structures and surgical phases, as well as providing real-time guidance on perfusion assessment during colorectal procedures [23,28,29]. One notable example, the Artificial Intelligence-Based Real-Time Microcirculation Analysis System (AIRAM), exemplifies how AI can enhance perfusion assessment during colorectal procedures [30].

Building on this foundation, deep learning (DL)—particularly convolutional neural networks (CNNs)—has demonstrated remarkable capabilities in analyzing complex visual and temporal data, such as laparoscopic video streams [28]. Surgical videos are inherently dynamic, featuring complex tool–tissue interactions that are challenging to accurately characterize using conventional analytical methods [26]. In this context, DL algorithms can recognize subtle patterns and extract subtle features from dynamic laparoscopic video streams that may be imperceptible to the human eye [31]. These systems can be further enhanced by integrating multimodal data sources, such as electronic health records, to generate individualized risk profiles for ALs in real time [32]. Applying this technology to the intraoperative detection of anastomotic leak could revolutionize surgical practice.

To date, most AI-assisted surgical tools have concentrated on assessing tissue perfusion and microcirculation—factors that are undoubtedly important for anastomotic healing [16]. However, relying solely on perfusion-based metrics may overlook the multifactorial and complex nature of AL development [33]. Elements such as anastomotic tension, technical precision, and patient-specific risk factors also significantly impact the likelihood of anastomotic failure and should be integrated into more comprehensive and multidimensional predictive models [34]. To truly improve intraoperative risk stratification, future AI systems must integrate multimodal data-driven solutions capable of capturing a broader spectrum of contributors to anastomotic failure. In this context, the ability to perform real-time analysis of intraoperative images using deep learning—providing accurate classifications with minimal processing time—could support real-time prediction and assist surgical decision-making throughout colorectal resection and anastomosis procedures, constituting a relevant novelty compared to the existing AI-assisted surgical tools.

In this proof-of-concept study, we propose the development of a DL-based predictive model for the intraoperative detection of anastomotic leaks during colorectal surgery. By analyzing laparoscopic and robotic video data, our aim is to identify high-risk anastomoses intraoperatively and in real time.

## 2. Materials and Methods

### 2.1. Patient Population and Study Design

For the development of this study, our group included a total of 26 patients whose surgeries were performed between June 2024 and December 2024 in three tertiary centers, namely in Portugal (Instituto Português de Oncologia de Lisboa Francisco Gentil [IPOLFG], Lisboa, Portugal), the United Kingdom (Royal Liverpool University Hospital [RLUH], Liverpool, United Kingdom), and Brazil (Hospital das Clínicas da Faculdade de Medicina de Ribeirão Preto [HCFMRP], São Paulo, Brazil). A total of 26 patients (IPOLFG, *n* = 12; RLUH, *n* = 6; HCFMRP, *n* = 8) were enrolled, from which 6 had ALs (IPOLFG, *n* = 2; RLUH, *n* = 2; HCFMRP, *n* = 2) and 20 had normal anastomoses (IPOLFG, *n* = 10; RLUH, *n* = 4; HCFMRP, *n* = 6).

Among the 26 patients included in this study, 25 underwent surgery for colorectal neoplasia and 1 for diverticulitis. Of these procedures, 6 were performed using a robotic approach, while 20 were conducted using a non-robotic technique. Regarding the type of surgery, 5 patients underwent right hemicolectomies, 3 left hemicolectomies, 4 proctosigmoidectomies, 6 segmental sigmoid resections, and 8 anterior rectal resections. The patients’ characteristics are presented in Table 1.

A total of 5356 still-frame images were used for the development and training of the CNN for the detection of anastomotic leak. The still-frame images were obtained during the surgical procedure, through decomposition of the procedure videos into frames, using VLC media player (VideoLan, Paris, France).

This study was performed after approval by the ethics committee of each study center. This was a retrospective non-interventional study, performed in accordance with the Declaration of Helsinki. Adequate omission of potentially identifiable patient information was assured, with each individual patient being assigned with a random number, guaranteeing data anonymization. The non-traceability of the data and adherence to the general data protection regulation (GDPR) was assured by a team with a Data Protection Officer (DPO).

### 2.2. Criteria for Definition of Anastomosis Leak

Each patient frame was classified either as having AL or as normal anastomosis. The definition of AL is still controversial given the lack of consensus among experts and the different grades of severity and AL-associated symptoms [3,35]. The diagnosis of AL was made in patients with either imagological, endoscopic, or surgical evidence of AL. Surgical evidence was considered in patients submitted to a surgical procedure due to poor clinical evolution with elevated levels of inflammatory markers. The criteria for reoperation were defined according to each center’s protocol. The diagnosis of a normal anastomosis implied hospital discharge without complications and at least 1 month of follow-up post-procedure. The definition of a normal anastomosis was established across all centers prior to the start of the study, as part of the predefined study protocol. Therefore, it was not subject to inter-institutional variability.

### 2.3. Development of the Deep Learning Model

A convolutional neural network (CNN) was used to automatically predict anastomotic leaks in colorectal surgeries. Model selection was performed by testing 4 different network architectures: EfficientNetB0, EfficientNetB7, ResNet50, and MobileNetV2 [36,37,38]. These deep learning models were developed to take as input an image with 3 channels and 224 pixels in height and width and output a 1D tensor of 1000 values, representing class scores for the 1000 categories in the ImageNet dataset, a comprehensive image dataset designed for object recognition. The model weights were initialized with the weights from the ImageNet dataset training. The feature extractor remained the same, and the fully connected layers from the classifier were removed and replaced by our own fully connected layers to adapt the model to the specific task. We used two blocks, each comprising a fully connected layer followed by a Rectified Linear Unit (ReLU) activation function and a dropout layer to prevent overfitting. The first fully connected layer had 512 neurons, and the last had 256. After these blocks, we incorporated a dense layer, whose size was determined using the binary classification (anastomotic leak (AL) or normal anastomosis (NA)).

The model output the probability of each frame being labeled as AL or NA, and the final classification was determined based on the one category with the higher probability. The model was developed to classify anastomotic leakage (AL) versus no anastomotic leakage (NA) based on imaging patterns observed in laparoscopic images. Heatmaps were also generated to visualize the frame features that contributed most to the model predictions.

Among the total dataset (*n* = 5356), 2007 frames were classified as anastomotic leaks, while the remaining 3349 frames were categorized as normal anastomoses. The dataset was divided into two main groups for this study: one for training and validation, and the other for testing. To maintain consistency, all frames from the same patient were kept together, following a patient-based split strategy. The training set, comprising 18 patients and 4962 frames, was used to train the model, while the validation set, with 4 patients and 181 frames, was used to fine-tune its hyperparameters. The testing set, with 4 patients and 220 frames, was used to independently evaluate the performance of the model.

The models were trained using Pytorch Lightning on an NVIDIA RTX A6000 graphics processing unit, alongside an AMD EPYC 7513 32-Core 2.6 GHz processor.

The Adam method was used to optimize the network parameters and set a fixed learning rate. The network was trained for a maximum of 200 epochs. Early stopping, a regularization strategy, was integrated into the training process to halt the training after 40 non-improving iterations. The metric used to assess improvement was the validation accuracy. The loss function used was the binary cross-entropy loss.

The model hyperparameters, including the initial learning rate (1 × 10^−5^), batch size (32), and dropout (0.4), were determined with a grid search, with the values considered presented in Table 2.

Data augmentation techniques were applied on the fly to the training data to increase data diversity and improve model generalization. This included horizontal flip, image cropping, and color jitter to change the images’ brightness, contrast, saturation, and hue.

### 2.4. Model Performance and Statistical Analysis

The CNN’s classification was compared to the corresponding label, and the evaluation and validation metrics were calculated. The primary outcome measures were the accuracy, F1-score, sensitivity, specificity, positive predictive value (PPV), negative predictive value (NPV), and area under the receiver operating curve (AUROC). Heat maps were also generated to enhance our understanding of the specific frame regions that contributed the most to the CNN’s prediction. Torchmetrics (1.7 version) was used for the statistical analysis.

## 3. Results

**Figure 1:**
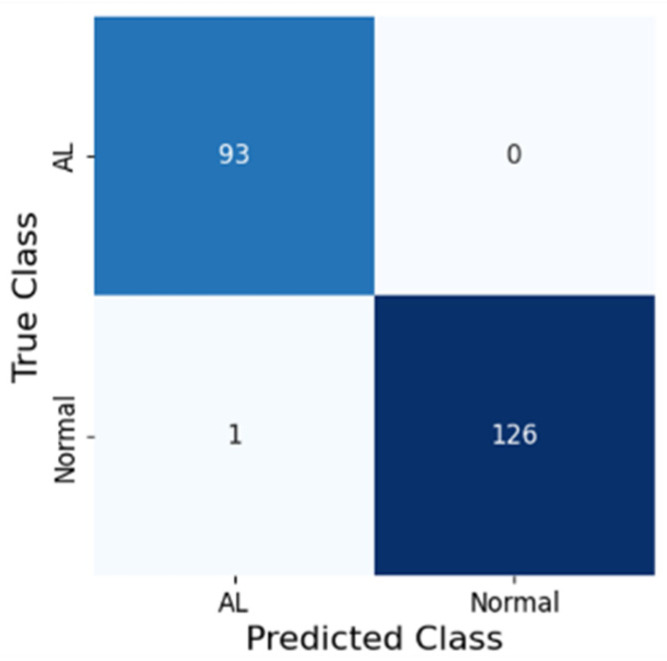
The confusion matrix between the model and gold-standard diagnostic criteria for anastomosis leakage diagnosis in the testing dataset. AL—anastomosis leakage.

Considering the total dataset, 2007 frames were classified as AL, while the remaining 3349 frames were categorized as normal anastomoses. Figure 1 showcases the confusion matrix between the model predictions and the gold standard of compound AL diagnostic criteria.

**Figure 2:**
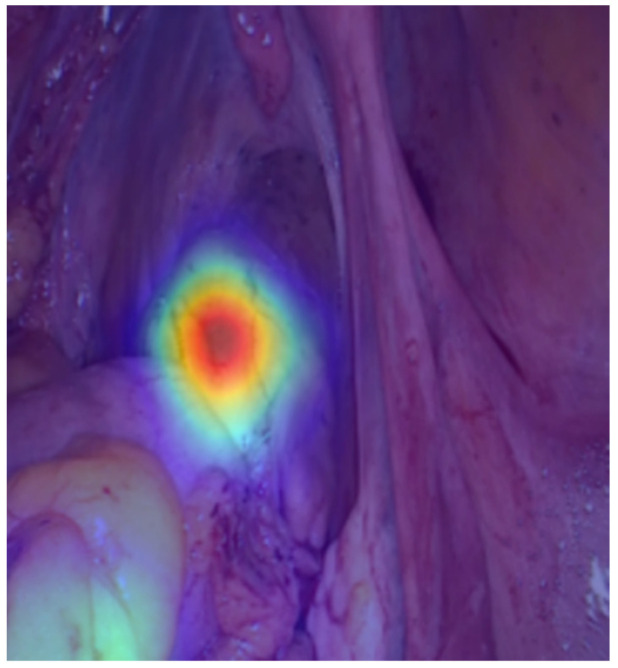
The heatmap generated by the deep learning model identifying the region of the image responsible for the anastomosis leakage prediction.

The metrics calculated for the best-performing model on the testing dataset are displayed in Table 3. The model achieved an accuracy of 99.5%, AUROC of 99.6%, sensitivity of 99.2%, specificity of 100.0%, PPV of 100.0%, and NPV of 98.9%. Heatmaps were generated as a method of explainable artificial intelligence, highlighting the most important region in the image for the model’s prediction (Figure 2).

## 4. Discussion

In this pioneer pilot study, our group developed a deep learning model for the prediction of AL in intraoperative laparoscopy frames. Besides the optimal diagnostic accuracy of the model, our group was also capable of developing an explainable artificial intelligence mechanism for the real-time identification of regions possibly translating into areas of AL. This study showcases AI’s potential for the real-time prediction of colorectal AL, creating the possibility of intraoperative diagnosis and decision-making, representing a potential paradigm change in colorectal surgery—one that may enhance patient safety, guide intraoperative strategy, and ultimately reduce the incidence and severity of AL-related complications.

The integration of AI into intraoperative decision-making could be a paradigm shift for AL prevention and treatment. AL’s definition is controversial, and a definitive consensus is lacking [3,35]. In fact, AL is commonly detected in patients with abdominal pain, fever, and elevations in inflammatory marker levels, with a radiological exam (CT with water-soluble enema) identifying a defect in the integrity of the intestinal wall at the anastomotic site [39]. However, AL can have several clinical presentations (from an absence of symptoms to septic shock), and its radiological manifestations can be subtle and nonspecific. Additionally, AL, specifically if detected later in the disease course, is associated with poor clinical outcomes and an impairment in quality of life [40]. In fact, AL results in a significant financial burden for both hospitals and society, with longer hospital stays and increased resource utilization [7].

A few works have focused on AI-assisted AL prediction or detection. In this context, preliminary AI-based scores and logistic regression models accurately identified risk factors for AL development, with early detection of high-risk patients, theoretically reducing the need for protective ostomy in low-risk patients [41,42]. Considering intraoperative detection and risk prediction, the majority of studies have focused on AI-enhanced microcirculation analysis [30,43]. Despite the promise of these machine learning tools, they are not available in clinical practice and are subject to significant heterogeneity. Therefore, our CNN is one of the first to automatically predict AL intraoperatively, with a frame processing rate that is suitable for real-time implementation. The possibility to accurately predict and identify AL intraoperatively would transform patient management, decreasing procedure-related complications and patient morbidity and mortality.

On the other hand, the role of derivative stomas (either colostomy or ileostomy) in AL detection, prevention, and management must be taken into consideration. In some cases, anastomotic leaks may remain subclinical due to the presence of a stoma, only becoming apparent at the time of stoma reversal—often when corrective management is more complex. In this context, there is still a lack of tools for the prediction of anastomosis leak risk in a patient in whom a protective stoma was constructed. This challenge could represent an opportunity for a deep learning model, which could support intraoperative decision-making, not only regarding the need for stoma creation but also in determining whether an anastomosis should be reinforced or revised. AI could be a solution for the identification of high-risk regions in an anastomosis for leakage development, specifically through explainable AI mechanisms such as heatmap generation. Nevertheless, its creation is dependent on developing a CNN based on a large number of patients with ALs, including those in whom a protective stoma was created and for whom available data until stoma closure can be analyzed. The present pilot study focused only on the identification of early AL, given the clinical relevance and associated costs, and, therefore, future studies will focus on a more complete model with a higher technology readiness level contemplating the prediction of both early and late AL.

Moreover, considerations must be made about the ethical and medico-legal concerns of AI implementation in colorectal surgery [44]. Firstly, AI implementation is dependent on a concise definition of responsibilities in case of error or misdiagnosis. In fact, and specifically considering intraoperative predictions of AL, the clinician/surgeon has the role of interpreting the AI prediction and deciding whether there is a need to modify the anastomosis or create a derivative stoma, or even not perform the anastomosis in a high-risk patient (due to age or comorbidities). Indeed, AI implementation during surgery is a critical area due to the implication of a misdiagnosis or wrong surgical decision. In this context, explainability is fundamental to assuring the comprehension of which area of the anastomosis was responsible for the model’s prediction [45]. Nevertheless, an explainable AI model is dependent on a large amount of data and the rigorous detection and notification of any bias in model development. In this context, data collection must respect the current legal framework, which is ultimately fundamental to the development of multimodal explainable AI models, which will stratify both patient risk and collected image information, guiding intraoperative surgical decisions.

Some methodological strengths of this study must be stated. Firstly, this pilot study resulted from data from three centers in three countries and on two continents. This study design was fundamental to ensuring the inclusion of patients from different clinical backgrounds and demographic contexts, reducing the risk of demographic bias in deep learning model development. Additionally, the training and testing datasets were divided with a patient split design, reducing the risk of model overfitting (as similar images from the same patients were exclusively in one of the datasets). With this objective, future studies may also include strategies such as cross-validation or an external validation cohort to further decrease this risk. Finally, the creation of an explainable AI mechanism showcasing the reasoning for model prediction is fundamental for ensuring greater surgeon confidence in the model’s decision and also for possibly guiding intraoperative decisions in areas with increased AL risk.

Nevertheless, a few limitations must be acknowledged. Firstly, this pilot study was based on a small dataset of 5000 frames from 26 patients undergoing colorectal surgery, which limits the study conclusions. The small dataset does not represent the number of procedures performed in each participating center, and the number of included cases was justified to create a balanced dataset of AL (which, fortunately, are rarer) and normal anastomosis cases. Future studies will incorporate a larger dataset with a multicentric and interoperable study design. Secondly, the study design was retrospective, and the CNN was developed using still frames. Further multicentric studies will focus on a real-time validation of the technology, integrating data that will allow for the prediction of both early and late ALs, with the possibility of real-time intraoperative feedback to the surgeon, augmenting surgical effectiveness and reducing the incidence of adverse outcomes.

## 5. Conclusions

In conclusion, this pioneer study showcased the first deep learning model for the prediction of early AL in laparoscopy images. Additionally, an explainable AI model was created to highlight the regions in the anastomoses that contributed the most to AL prediction. This study marks the first steps in AI-assisted laparoscopy, in which AI models will influence both intraoperative diagnosis and decision-making, being an additional help to modify patient outcomes and reduce the risk of postoperative AL.

## Figures and Tables

**Table 1:** Patient baseline characteristics.

	Patients (*n* = 26)
**Age, years (SD)**	62.3 (11.5)
**Sex, *n* (%)**	
Female	13 (50.0)
Male	13 (50.0)
**Surgery, *n* (%)**	
Right hemicolectomy	5 (19.2%)
Left hemicolectomy	3 (11.5%)
Proctosigmoidectomy	4 (15.4%)
Segmental sigmoid resection	6 (23.1%)
Anterior rectal resection	8 (30.8%)
**Study Center, *n* (%)**	
Instituto Português de Oncologia de Lisboa Francisco Gentil, Portugal	12 (46.2)
Royal Liverpool University Hospital, United Kingdom	6 (23.1)
Hospital das Clínicas de Ribeirão Preto, Brazil	8 (30.8)
**Surgical Indication**	
Neoplasia, *n* (%)	25 (95.2)
Diverticulitis, *n* (%)	1 (4.8)
**Robotic Surgery, *n* (%)**	6 (23.1)
**Anastomotic Leak, *n* (%)**	6 (23.1)

SD—Standard deviation.

**Table 2:** The hyperparameter tuning values used in the grid search.

Hyperparameter	Possible Values		
**Learning Rate**	1 × 10^−7^	1 × 10^−6^	1 × 10^−5^
**Batch Size**	16	32	64
**Dropout**	0.3	0.4	0.5

**Table 3:** Performance metrics on the testing dataset set. PPV—positive predictive value. NPV—negative predictive value. AUROC—area under the receiver operating characteristic curve.

Accuracy (%)	Sensitivity (%)	Specificity (%)	PPV (%)	NPV (%)	AUROC (%)
99.5	99.2	100.0	100.0	98.9	99.6

## Data Availability

Additional data are available upon reasonable request.
